# Factors influencing length of survival in ambulatory palliative care - a cross sectional study based on secondary data

**DOI:** 10.1186/s12904-021-00762-x

**Published:** 2021-05-17

**Authors:** Johannes Just, Marie-Therese Schmitz, Ulrich Grabenhorst, Thomas Joist, Kirsten Horn, Bettina Engel, Klaus Weckbecker

**Affiliations:** 1 Institute of General Practice and Interprofessional Care Witten/Herdecke University, Alfred-Herrhausen-Straße 50, 58453 Witten, Germany; 2 Department of Medical Biometry, Informatics and Epidemiology (IMBIE), University Hospital Bonn, Sigmund-Freud-Str. 25, 53127 Bonn, Germany; 3 VSTN e.V. (Association of SAPV Teams in North Rhine), Venloer Straße 40, 41751 Viersen, Germany; 4 Division of General Practice, Department of Health Services Research, Carl von Ossietzky University of Oldenburg, 26129 Oldenburg, Germany

**Keywords:** Palliative Care, Survival, Ambulatory Care, non-cancer disease

## Abstract

**Background:**

Quality of life and patient self-determination are key elements in successful palliative care. To achieve these goals, a robust prediction of the remaining survival time is useful as it can provide patients and their relatives with information for individual goal setting including appropriate priorities. The Aim of our study was to assess factors that influence survival after enrollment into ambulatory palliative care.

**Methods:**

In this cross-sectional, multicenter study (n = 14 study centers) clinical records of all palliative care patients who were treated in 2017 were extracted and underwent statistical analysis. The main outcome criterion was the association of survival time with clinical characteristics such as age, type of disease, symptoms and performance status.

**Results:**

A total of 6282 cases were evaluated. Median time of survival was 26 days (95 % CI: 25–27 days). The strongest association for an increased hazard ratio was found for the following characteristics: moderate/severe weakness (aHR: 1.91; 95 % CI: 1.27–2.86) Karnofsky score 10–30 (aHR: 1.80; 95 % CI: 1.67–1.95), and age > 85 (aHR: 1.50; 95 % CI: 1.37–1.64). Surprisingly, type of disease (cancer vs. non-cancer) was not associated with a change in survival time (aHR: 1.03; 95 % CI: 0.96–1.10).

**Conclusions:**

In this cross-sectional study, the most relevant predictor for a short survival time in specialized ambulatory palliative care was the performance status while type of disease was irrelevant to survival.

## Background

Quality of life and patient self-determination are key elements in successful palliative care [1, 2]. These goals can be helped by a robust prognosis as it can provide patients and their relatives with information for individual goal setting including appropriate priorities and expectations of care and can help health-care professionals with patient-centered clinical decision making [3–7].

Despite the importance of prognosis for successful palliative care, clinicians are sometimes reluctant to give a precise prognosis of medical outcomes, especially death [8, 9]. Physicians reported poor training, lack of data and difficulties in formulating and communicating as major problems in delivering a prognosis [10]. Furthermore, they were worried that patients and colleagues may judge them poorly when the prognosis is incorrect. Their worries are understandable, as prognosis in terminally ill patients is complicated and often inaccurate [11].

Although data on predictive factors for an impending death are limited, some clinical factors associated with an increased risk for an impending death have been identified and may help clinicians to improve the accuracy of their prognosis. They include symptoms associated with cancer anorexia-cachexia syndrome, dyspnea and delirium but were mostly investigated in cancer patients [5, 12]. A retrospective cohort study from the UK with 4650 patients showed that days in palliative care were especially limited for those patients with non-cancer disease and older age [13].

While a reliable prediction to the day of death is yet out of reach, identifying further patterns associated with an impending end-of-life situation may be a next step to help physicians to improve the quality of prognosis, steering them away from existing coping strategies like avoidance, optimism and vagueness [8–11]. In order to do so, more data from large palliative care patient collectives with cancer and non-cancer disease is needed.

Our aim was to analyze clinical data from ambulatory palliative care providers in Germany in order to identify clinical factors that influence survival rates of terminally ill-patients with cancer and non-cancer disease, including basic characteristics like age, burden of symptoms and performance status at the time of submission to palliative care.

In Germany, patients with a life-limiting disease and a high symptom burden can be referred to a high-quality, visit-based ambulatory palliative care system called SAPV (“Spezialisierte Ambulante Palliativ-Versorgung = spezialiced ambulatory palliative care), which delivers specialised medical and nursing care services and is covered by the statutory health care system. The main aim of SAPV is to enable these patients to remain in their home or family environment despite complex treatment needs. SAPV is designed to deal with disease-related crisis situations that might otherwise lead to undesired and stressful hospital admissions in patients where curative treatment is no longer an option. To do so, the service providers must have appropriate training and must guarantee 24-hour availability seven days a week [14, 15].

## Methods

### Study design

We conducted a retrospective cross-sectional analysis from anonymized routine treatment data aiming to identify clinical factors that influence survival rates of terminally ill-patients with cancer and non-cancer disease.

### Setting and locations

Data was retrieved from 14 SAPV providers in the administrative “Nordrhein-region”, Germany. Each provider is certified for a defined geographic area. Cumulatively, those 14 providers cover appx. 5 million (52.6 %) of the 9.5 million inhabitants in the “Nordrhein-region” [16].

### Data sources and measurement

The patient data was recorded by the SAPV providers in the Digital Documentation System Palliative Care® (ISPC) by the company SmartQ-Softwaresysteme GmbH or PalliDoc® by the company StatConsult (Gesellschaft für klinische und Versorgungsforschung mbH) as part of routine case documentation.

Data extraction was coordinated by the Association of SAPV-Teams in North Rhine e.V. (VSTN = Verbund der SAPV-Teams Nordrhein e.V.) of which all participating data providers are a member. The entire patient information of the electronic files of the 14 providers was extracted from ISPC® and PalliDoc® in anonymised form on the patient as well as the provider level. Merging and data quality management was performed by StatConsult, a software developer and contract institute for tasks in clinical research, development and health services research.

### Ethical considerations

The requirements of the current version of the Declaration of Helsinki have been observed. The responsible ethics committee of the North Rhine Medical Association evaluated the project and issued no objection (process number 57/2017).

### Participants and case definitions

In order to distinguish cancer from non-cancer patients, all patients with an admission diagnosis or main diagnosis according to ICD-10 C00 - D48 were labeled “cancer patients”. A co-diagnosis of cancer was considered to be secondary for enrollment into SAPV (e.g. admission diagnosis dyspnea, primary diagnosis heart failure, secondary diagnosis prostate cancer).

The dataset includes all patients who were under treatment in 2017. This means that cases that started before 2017 or continued into 2018 were also part of the dataset.

SAPV is not always continuous and can be paused or completely ended for various reasons e.g. hospitalization, transfer to hospice or improvement of symptom burden due to therapy.

About one third of the patients in our dataset showed several treatment periods varying in length of treatment as well as gap-period. In order to include these patients in the analysis, we decided to merge cases in which the treatment gaps did not exceed 13 days. The clinical rationale was that short gaps mostly derived from hospital visits for specialised procedures such as palliative chemotherapy or erythrocyte transfusion and were therefore caused by accounting requirements and not because patients ceased to have an indication for ambulatory palliative care. The 13 days cut-off was chosen based on expert opinion as well as coverage of 75 % of cases with a treatment gap. Naturally, much larger treatment gaps occurred in a minor amount of cases which we attributed to an overall improvement in symptom burden after an initial crisis situation, which enabled the patient to return into general primary care for a period of time. In these cases, only treatment periods after the last treatment-gap > 13 days were included. We performed a sensitivity analysis to control for relevant differences between patients with and without a treatment gap > 13 days.

### Quantitative variables and statistical analysis

Descriptive statistics were used to present characteristics of the study population. The characteristics included: Gender, age, Karnofsky-score, pain, dyspnea, confusion, loss of appetite, weakness, emesis, nausea. Categorical variables were summarized by absolute and relative frequencies, e.g. the Karnofsky score, a performance scale used to quantify cancer patients’ general well-being and activities of daily life ranging from 0 (death) to 100 (perfect health) was categorised as low when the patient was bedridden (10–30). Symptoms were assessed by palliative care specialists using 5- and 10-point Likert-Scales. In an attempt to harmonize reporting between the different teams, they were redistributed upon data extraction and grouped as none, mild, moderate and severe. Because of the distribution pattern, moderate and severe symptoms were later pooled for statistical analysis as it was clinically acceptable and led to a similar group sizes. Kaplan-Meier estimates were used to visualize the observed time to death in different patient groups (cancer/non-cancer, dichotomized Karnofsky-score and age). Additionally, the median survival time including 95 % confidence intervals (CI) was determined. In order to further analyse the association between the survival time of the patients and several variables (cancer/non-cancer, Karnofsky-score, age, gender and symptoms such as pain, dyspnea, confusion, loss of appetite, weakness, emesis and nausea), a Cox proportional hazard model was used and hazard ratios (HR) as well as adjusted hazard ratios (aHR) with 95 % CI were reported. The multivariable Cox model included all aforementioned variables. In regression analysis, missing values within the independent variables were assigned to the most frequent category only when the amount of missing values was below a threshold of 5 % (only for cancer/non-cancer groups and gender). Otherwise missing values were summarized into a separate category. All analyses were carried out using R (version 4.0.2) and SAS 9.4. The documentation of the analysis is based on the recommendations of the STROBE statement [17].

## Results

Within the year 2017, a total of 6828 patients were treated by 14 SAPV teams in North Rhine-Westphalia of which 75.3 % (n = 5141) died within their treatment period which amounts to roughly 9 % of the total annual death toll in the region [18]. Most patients had one single treatment period prior to death (67.9 %), a further 21.5 % of patients had two or more consecutive treatment periods with only short gaps (< 13 days) that were therefore combined for analysis. The number of cases where prior treatment periods were excluded because of a longer treatment gap (> 13 days) was 10.6 %. Patients with no or short treatment gaps were slightly older and showed a higher rate of cancer disease compared to those with longer treatment gaps (median age 75.3 vs. 71.8 years; rate of cancer-disease 74.7 vs. 80.0).

An overview of the basic sociodemographic data as well as basic functionality indicators and symptom burden of the complete study population is given in Table 1:

**Table 1:** Characteristics of the study population divided by cancer and non-cancer disease

Variable	Total (*n* = 6828)		Cancer^a^ (*n* = 5140)		Non-cancer^a^ (*n* = 1665)	
Gender	n	%	n	%	n	%
Male	3337	48.9	2632	51.3	696	41.9
Female	3482	51.0	2502	48.7	966	58.1
Missing	9	0.1	6		3	
Age, years						
<65	1409	20.6	1257	24.5	148	8.9
65–74	1416	20.7	1203	23.4	208	12.5
75–84	2349	34.4	1820	35.4	522	31.4
≥ 85	1654	24.2	860	16.7	787	47.3
Karnofsky score						
10–30	2124	31.1	1242	42.1	879	82.8
40–100	1892	27.7	1708	57.9	182	17.2
Missing	2812	41.2	2190		604	
Pain						
No	1155	16.9	883	22.2	267	21.8
Mild	1723	25.2	1339	33.6	382	31.2
Moderate/severe	2341	34.3	1758	44.2	575	47.0
Missing	1609	23.6	1160		441	
Dyspnea						
No	1812	26.5	1374	32.6	431	33.2
Mild	1821	26.7	1473	34.9	347	26.7
Moderate/severe	1895	27.8	1372	32.5	520	40.1
Missing	1300	19.0	921		367	
Confusion						
No	2672	39.1	2356	59.9	313	24.8
Mild	1040	15.2	812	20.7	226	17.9
Moderate/severe	1489	21.8	763	19.4	721	57.2
Missing	1627	23.8	1209		405	
Loss of appetite						
No	670	9.8	517	11.6	152	11.3
Mild	1002	14.7	826	18.5	175	13.0
Moderate/severe	4146	60.7	3113	69.9	1021	75.7
Missing	1010	14.8	684		317	
Weakness						
No	48	0.7	43	0.9	5	0.4
Mild	566	8.3	511	11.2	54	3.9
Moderate/severe	5368	78.6	4017	87.9	1334	95.8
Missing	846	12.4	569		272	
Emesis						
No	4033	59.1	2986	77.4	1040	87.5
Mild	595	8.7	506	13.1	89	7.5
Moderate/severe	427	6.3	368	9.5	59	5.0
Missing	1773	26.0	1280		477	
Nausea						
No	3097	45.4	2181	53.4	911	75.6
Mild	1301	19.1	1108	27.1	191	15.9
Moderate/severe	899	13.2	796	19.5	103	8.5
Missing	1531	22.4	1055		460	

^a^Relative frequencies refer to non-missing cases only. There were n = 23 patients with missing data on diagnoses (0.3 %)

In general, patients with non-cancer disease showed a higher burden of symptoms with increased rates of moderate and severe confusion, dyspnea, loss of appetite and weakness, while patients with cancer disease showed increased rates of emesis and nausea. However, the arguably most distressful symptom, pain, was almost equally distributed between both groups.

**Fig. 1:**
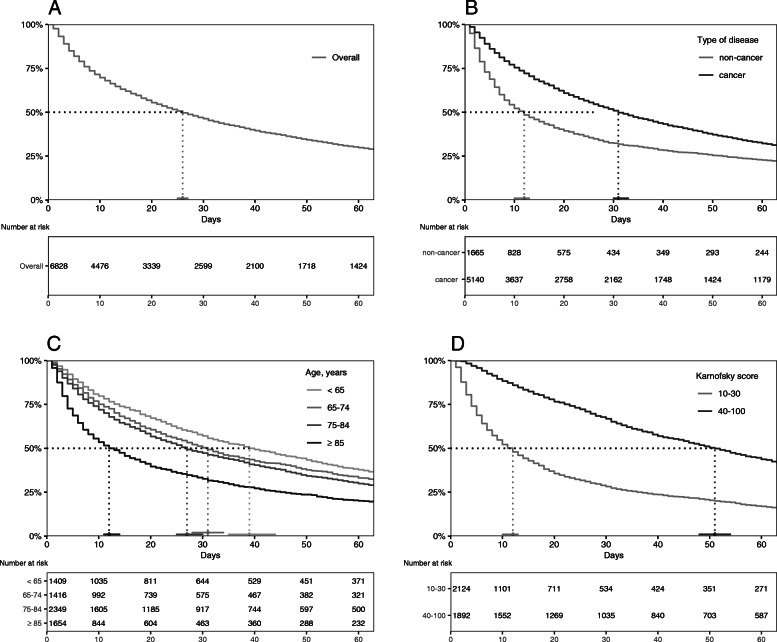
Kaplan-Meier estimates (up to 60 days) and median survival for different patient groups

The overall median survival based on the treatment periods was 26 days, meaning that 50 % of patients died within the first 26 days (95 % CI: 25–27 days) while 25 % died within 8 days and 75 % within 75 days. A visual representation of the Kaplan-Meier estimate up to 60 days is given in Fig. 1, Curve A.

Additionally, Kaplan-Meier Curves for potentially relevant influencing factors on survival were created (Fig. 1 Kaplan-Meier Curves B, C, D). The choice of influencing factors was literature based [5, 12, 19].

Figure 1 legend: missing values are not shown (Fig. 1 **a**: n = 0 (0.0 %); Fig. 1**b**: n = 23 (0.3 %); Fig. 1 **c**: n = 0 (0.0 %); Fig. 1**d**: n = 2812 (41.2 %)).

As we suspected a strong overlap between several characteristics (e.g. “age” and “Karnofsky-Score”), we used a cox proportional hazard model in order to adjust for the individual effects of influencing factors. Thus, we were able to show that the functional status as represented by weakness and the Karnofsky-score upon enrollment in SAPV was the most relevant prognostic factor, regardless of type of disease. All hazard ratios are shown in Table 2.

**Table 2:** Cox-hazard regression model

Variable	Unadjusted HR	95 %-CI	Adjusted HR	95 %-CI
Gender^a^				
Male	Reference	–	Reference	–
Female	0.95	0.90–1.00	0.88	0.83–0.93
Age, years				
<65	Reference	–	Reference	–
65–74	1.18	1.08–1.28	1.09	1.00–1.19
75–84	1.25	1.16–1.35	1.15	1.06–1.24
≥ 85	1.82	1.68–1.98	1.50	1.37–1.64
Diagnosis^a^				
Cancer	Reference	–	Reference	–
Non-cancer	1.45	1.36–1.54	1.03	0.96–1.10
Karnofsky performance score				
40–100	Reference	–	Reference	–
10–30	2.29	2.13–2.46	1.80	1.67–1.95
Missing	1.44	1.35–1.55	1.21	1.12–1.31
Pain				
No	Reference	–	Reference	–
Mild	1.08	0.98–1.17	1.02	0.94–1.12
Moderate/severe	1.31	1.21–1.42	1.14	1.05–1.25
Missing	1.39	1.27–1.52	0.96	0.86–1.07
Dyspnea				
No	Reference	–	Reference	–
Mild	1.00	0.92–1.07	1.01	0.93–1.09
Moderate/severe	1.26	1.17–1.36	1.16	1.08–1.25
Missing	1.67	1.54–1.82	1.18	1.04–1.35
Confusion				
No	Reference	–	Reference	–
Mild	1.35	1.25–1.47	1.10	1.01–1.20
Moderate/severe	1.88	1.75–2.02	1.33	1.23–1.44
Missing	1.66	1.54–1.79	1.15	1.02–1.30
Loss of appetite				
No	Reference	–	Reference	–
Mild	1.02	0.90–1.15	1.02	0.90–1.15
Moderate/severe	1.70	1.54–1.87	1.44	1.30–1.60
Missing	2.34	2.08–2.63	1.31	1.09–1.56
Weakness				
No	Reference	–	Reference	–
Mild	1.20	0.80–1.82	1.56	1.03–2.37
Moderate/severe	2.20	1.48–3.29	1.91	1.27–2.86
Missing	3.81	2.53–5.72	2.78	1.80–4.28
Emesis				
No	Reference	–	Reference	–
Mild	1.15	1.05–1.27	1.02	0.91–1.13
Moderate/severe	1.26	1.13–1.40	1.13	0.99–1.29
Missing	1.43	1.34–1.53	0.98	0.86–1.12
Nausea				
No	Reference	–	Reference	–
Mild	1.01	0.94–1.09	0.96	0.89–1.05
Moderate/severe	1.10	1.01–1.20	0.99	0.89–1.11
Missing	1.58	1.47–1.69	1.21	1.05–1.39

^a^Female includes n = 9 patients with missing values on gender; cancer includes n = 23 patients with missing values on diagnosisref. reference; *HR* hazard ratio; *CI* confidence interval

## Discussion

Our data shows that patients with non-cancer disease were admitted to SAPV with a much lower performance status, which in turn was the strongest predictor of a short life expectancy. The burden of symptoms upon enrollment into SAPV was at most slightly higher in patients with non-cancer disease but the most important symptom in palliative care, pain, was equally distributed between patients with and without cancer disease.

The strength of this analysis includes the multi-center approach and the high number of cases. To our knowledge, it is the largest collective of cancer and non-cancer patients in ambulatory palliative care that has been studied regarding survival rates to date.

In terms of generalizability, the multi-center approach makes it representative for the Nordrhein-region and relatively representative for SAPV in Germany as a whole. As data was extracted from health care data instead of insurance data, it covers the whole population and doesn’t exclude patients with private insurance. SAPV is a well-funded and well received instrument of care that is accessible through statutory health insurance as well as private insurance and therefore is theoretically accessible to every German citizen. So while the actual survival rates of patients may not be comparable to patients who receive a different type of care in other parts of the world, we assume that the influencing factors for a decrease in survival time may be generalizable to an ambulatory care setting for patients with a life-limiting disease and a high symptom burden .

In terms of validity, we expect the data to be of decent quality, as it represents clinical data on symptoms and patient characteristics collected by trained palliative care physicians and nurses. A clear weakness of the data is the high rate of missing values. This may be due to the circumstance that different SAPV providers have different documentation standards. For instance, some may assess the presence of confusion only in patients where it is clinically expected while others may do it as a default.

In terms of reliability, we expect the data to be comparable to prior years, but rather drastic changes are possible e.g. when financial compensation mechanisms get changed or during a pandemic situation.

The general characteristic and the symptom burden of the patient collective in our analysis was consistent with prior studies in the field. Patients with non-cancer disease were generally older, had a poorer performance status but showed no differences in pain intensity as compared to cancer patients [19, 20].

Our findings on time of survival for patients with and without cancer-disease are consistent with other studies in the field and show a significantly reduced survival time of patients with non-cancer disease [13]. As shown in Fig. 1 using a Kaplan-Meier curve, type of disease (cancer vs. non-cancer), Karnofsky-Score (10–30 vs. 40–100) and age (< 65 vs. >85 years) were all associated with significant differences in length of survival. As these factors were very likely interdependent, we adjusted for several variables in a Cox proportional hazard model. The model however showed that the type of disease (cancer vs. non-cancer) had no influence on length of survival (aHR: 1.03; 95 % CI: 0.96–1.10). The strongest association for an increased hazard ratio was found for the following characteristics: moderate/severe weakness (aHR: 1.91; 95 % CI: 1.27–2.86), Karnofsky score 10–30 (aHR: 1.80; 95 % CI: 1.67–1.95), and age > 85 (aHR: 1.50; 95 % CI: 1.37–1.64). Studies based on hospitalized cancer patients have identified similar risk factors in the past, mainly low performance scores and cancer-anorexia syndrome but also confusion and dyspnea which all correspond well with our results [5, 12]. These results are an important addition to the existing knowledge base and clearly show that performance status and age are the most important predictors of a low life-expectancy in ambulatory palliative care.

It is a common assumption in palliative care, that patients with non-cancer disease are enrolled into palliative care too late or at least later than patients with cancer-disease. Our data however doesn’t generally support this assumption. While non-cancer patients do spend less time in ambulatory palliative care, this is clearly due to their lower performance status and higher age upon enrollment. It may be possible that non-cancer patients just develop palliative care needs later in the last phase of life, an assumption that is supported by the very similar distribution of pain severity in cancer and non-cancer patients upon enrollment. Patients with non-cancer disease show a much higher rate of confusion but this is most likely due to their higher age, an assumption that is supported by the drop in the HR for confusion after adjustment for age and other factors. We therefore hypothesize that patients with non-cancer disease may not be generally referred to SAPV too late in relation to pain. It remains unclear if the lower performance status upon enrollment is a sign of delayed enrollment or a co-factor of the non-cancer patients’ higher age. Qualitative research into the functional burden on quality-of-life of cancer and non-cancer patients in SAPV might be helpful to answer this question.

## Conclusions

Performance status is the most important influencing factor for a decrease in survival time in patients in ambulatory palliative care. Old age and the presence of several symptoms including loss of appetite and confusion are secondary influencing factors for a decreased survival time. Survival time is not influenced by the type of disease (cancer vs. non-cancer). This should be kept in mind when discussing prognosis with a patient and his or her family. Albeit it should be noted, that survival time is only one side of the medal, the other being quality-of-life.

We found no evidence that patients with non-cancer disease who do get admitted to SAPV, are admitted too late. They do not suffer from pain more than patients with cancer disease upon enrollment. We therefore assume that the SAPV may be rather well timed for those patients with non-cancer disease who receive it.

Further investigation should focus more on outcome criteria (e.g. quality-of-life) and should correlate those outcome criteria with length of survival in order to better investigate the important balance of life-expectancy and quality-of-life.

Declarations.

GmbH, Palliativteam SAPV RheinErft GmbH, Regionales Gesundheitsnetz Leverkusen eG, SAPV Team Solingen GmbH, SAPV Wuppertal GmbH, SAPV-Krefeld GbR, SAPV-Team NoPaiN GmbH.

## Data Availability

The datasets generated and analysed during the current study are available from the corresponding author upon reasonable request.
